# Atlanta Society of Medicine

**Published:** 1899-09

**Authors:** 


### SOCIETY REPORTS.

### ATLANTA SOCIETY OF MEDICINE.

ATLANTA, GA., June 15, 1899.

*Regular Meeting of the Atlanta Society of Medicine.*

The President called for report from the committee appointed on May 18th in regard to means of controlling contagious diseases.

Dr. Kime, of that committee, stated that they had tried to have a meeting with the Board of Health, but had failed to get them together. However, the committee had a meeting and discussed the question, and decided that it was best to have a medical health officer and also a bacteriological laboratory; the health officer to be on the Board of Health. That they thought it best to get the Society together and have a discussion and get up ideas as to the best plan, and then get a harmonious action of the Board of Health, and then go to the Council. Want to get the medical profession to stand by whatever action the committee may decide upon. That he had talked with the different members of the Board of Health, and they all seemed to be in favor of the plan, and the next thing will be to get the necessary appropriation from the Council. That he had requested Dr. Stirling and Dr. Robinson to write out their ideas on the subject.

Dr. Stirling read as follows:

"DUTIES OF MEDICAL HEALTH OFFICER.

"1. Attend at Health office at certain hours to interview inspectors and be accessible to persons desirous of seeing him.

"2. Should personally inspect premises when inspector's report is disputed; when any legal action is contemplated; when illness supposed to be due to condition of premises; in all cases of complaint of nuisance caused by noxious trades; occasionally inspect premises used as public lodging houses, if any.

"3. Should receive every morning reports from the inspectors concerning the work of the previous day.

"4. Should narrowly watch the condition of the water supply.

"5. Should inquire into state of premises in which an infectious disease exists; insist upon disinfection at the proper time. If patient is a school-child, the medical health officer should see that school authorities are informed of illness, and that, where necessary, other members of family are not allowed to go to school. Should remove compulsorily to hospital, where legally permitted, such infectious cases as cannot be treated properly at home.

"6. Should keep proper books showing a statement of his work.

"7. Should report at stated intervals in writing to the Board of Health upon the condition of the city so far as it comes under his jurisdiction.

"8. Should do such bacteriological work for the Board of Health as may be arranged, and at the terms arranged."

Dr. Robinson stated that there was one other thing which Dr. Kime intended to speak of, and that was for each individual member of the Society to use his influence with the Council to get this passed. That the family physician of each member of the Council would probably have most influence with him. That every member of the Society should talk and create a public opinion as much as possible in favor of a medical inspector.

*Dr. J. C. Johnson:* I was not here when this movement was introduced, but I infer the nature of it from the report of the committee, and have long thought that some provision should be made by the city with the view of accomplishing the ends mentioned by the committee. I think by all means we ought to have more rigid inspection and more careful attention given to contagious diseases and more careful attention to the disinfection of premises, especially with reference to the time of disinfection. Some provision should be made for those physicians who are not able to make bacteriological examinations, or patients who are not able to pay for it. In fact, the spirit of the movement I am in full accord with, and in favor of creating a sentiment of public opinion and influencing the members of the Council toward establishing this.

*Dr. Hardon:* Of course we all recognize the importance of the objects which have been specified, and in a city the si ze of Atlanta it is of the utmost importance that every sanitary protection should be taken. I presume the committee have given it sufficient thought to enable them to formulate their ideas more correctly than others who have not given the same amount of thought to it. I would be the last man to throw water on it, or anything like this, but would ask, what is the Board of Health for? and do they not about cover the ground mentioned by the committee? It seems to me that this comes within their jurisdiction. I may be mistaken about this, and if they do not, we ought to have a health officer. There ought to be some one to see that these sanitary precautions are carried out. However, I don't envy the man who has the job. Again, it will not be an easy matter to carry this out, for a body of men which cannot be influenced to fill up the wells in the city can hardly be gotten to support this. We certainly should use every effort to carry this out.

*Dr. Duncan:* I have thought about this thing, and I am not very much in sympathy with it. I think every doctor should look after the health of his patients and the interest of the public schools, and I think that the Board of Health, with the inspectors and the physicians, should be able to cover the ground without any extra expense. It would give a place for a man who is not doing very much. To get a man to fill that place he would have to be very competent and necessarily would have to have a good salary, and therefore I am not in sympathy with it.

*Dr. Kime:* We don't want to go into this thing unless we have the sympathy and support of the Society; unless we can stir up the men sufficiently to let them know what are the duties of the Board of Health, and that the ground is not covered and infectious diseases are not controlled. Unless we do this and have thorough cooperation we cannot get the Council to act upon it. We want to get the physicians outside stirred up, as well as the members of the Society.

It is not presumed that any member of the Board of Health is to go out and make diagnosis of a disease. There are not twelve men in Atlanta who can take a microscope and make a diagnosis, and cannot make an examination of a specimen of well water. What we mean by a medical inspector will be a man who will be under the Board of Health and as soon as any physician has any case which he thinks is diphtheria, scarlet fever, or small pox, etc., whether or not he has made a diagnosis, it will be the duty of the inspector to see whether the family is carrying out the necessary precautions, and also to notify the authorities. For instance, there is a case in which there is doubt as to the cause and the children should be kept at home, which is not usually done, the inspector would see that this is enforced. You can sometimes find a dozen cases with croup and membranous croup that have gone to school, also cases of scarlet fever where the children have been allowed to attend church before the card has been taken off the door. We want a man to see that the proper precautions are carried out, and to instruct the family as to what is to be done, as there are very few doctors who know what should be done, that is, as to what the nurses and different ones should do, the number of days the patient should remain in the room, the number of days that must elapse before the disease can be conveyed to others. I have been informed by teachers in the public schools that children who have been under the care of good physicians have been allowed to come back to school with the skin peeling off their fingers after an attack of scarlet fever. This condition of affairs would not exist if we had proper control over this matter. As I have said before, physicians who will allow patients to go out this way should not be allowed to practice. There are men who will cater to the whims of the patients, and will be persuaded to let the patient out before their judgment tells them they should. We want to get a man who understands thoroughly the diagnosis and duration of these infectious diseases, and if a physician is in doubt this inspector will visit that patient and take specimens and make cultures, etc., and assume the responsibility of the diagnosis. I know of one case where a diagnosis of scarlet fever was made and the card was put up, and some time after this another physician was called in and he decided it was not scarlet fever because the other had decided that it was. This case brings up the necessity of having rigid inspections. This health officer would be paid by the city and therefore does not cater to the patient. When it is questionable as to whether the patient should be allowed to go out the expert is called to examine and finds should not be dismissed he holds the patient over a while. We need that check right here in the city. These are some of the reasons why a health officer is needed. We also need a man who is able to make bacteriological tests, etc. Very frequently the water is all right so far as the chemical tests, but the bacteriologist will find things that should not be there. We want an inspector and a laboratory to carry out tests. This is what the Board of Health wants.

Another thing, of course, we do not want this matter to get into politics. The inspector should be left free to exercise his knowledge and not to be affected by politics. This man should be selected by this body and not controlled by the public, and then confirmed by the Council, and should be elected for a length of time, at least three to five years, so as to become thoroughly familiar with the work, and the inspectors of the city should be under him.

*Dr. Johnson:* If the spirit of that movement is in strict accord with Dr. Kime's remarks, I am in part in favor of and in part opposed to it. I agree with him that many children who have scarlet fever particularly, and diphtheria are allowed out of the house and on the street before they should do so. But when we begin to agitate the question of contagiousness we have to recognize that there is a great discrepancy of opinion as to this contagion. For instance, where the desquamation is found between the fingers. I have seen cases of seven weeks after the fever, and I was fully convinced that the scales which came off at this time would bear no contagion; so I am not ready to condemn those physicians who see fit to turn them aloose at a time when I would not do so. I do not deny that some are turned loose too soon, and I still say that it should be looked into.

There is another thing: to appoint a health officer having the authority that Dr. Kime would confer upon him would interfere somewhat with the individual rights of members of the profession in the city. I take it for granted that every member of this Society knows enough about the danger of contagious fevers to take the necessary precautions, and it is his duty to report all cases, and when he is in doubt to call in consultation. If I should meet with a case to-morrow where I suspected scarlet fever I would isolate the case, and then if I was still in doubt, where there was cause for hurry, I would call in another physician. We can find among the profession in Atlanta men who can aid in diagnoses as well as a man appointed for that purpose.

Again, Dr. Hardon asks the purpose of the Board of Health. This is an assertion as well as an interrogation. The Board of Health is to look after the health of the city, and this includes water, sewers, etc. I understand that this officer was simply to examine the water, milk, etc., and assist in the work. I do not agree with Dr. Kime that there are not twelve men in Atlanta capable of making bacteriological diagnosis. I am afraid he under-estimates the intelligence of the profession, and there is no man who will submit to any officer of the Board of Health coming in and taking charge of his case. I am opposed to any movement looking to that end, as you cannot interfere with the individual rights of the physician. Along this line I sometimes even doubt the expediency of consultation, and to have a physician come in and say you have to do so and so, this is not practicable, and could not be carried into effect, as it would meet with opposition. I understand this officer was to aid the Board of Health and make the bacteriological examinations, and to respond where he was called, and if necessary, supervise the disinfection. However, I have no criticism to make of the method of disinfection in Atlanta. We have a Board of Health to direct the inspectors, and if we have an officer under the Board of Health he can supervise the work. Hovever, we sometimes have several cases to be disinfected at one time, and have to wait because we have not enough inspectors, and if we cannot have enough inspectors, how can we have enough officers to superintend it. I think there is an impracticable feature about the movement. If we can have an officer for the practical work I am in favor of the sentiment. I know there are some children who go out too soon after having contagious diseases, but the physician is required to make report of these cases to the Board of Health, and if he has no more sense than to turn a child loose a week before he should, then his diploma should be taken from him. However, as I said before, there is a great difference of opinion as to the contagion after having a disease. Take a child who has had scarlet fever. Very few children contract scarlet fever during the attack of the fever, but it is during the desquamative stage. However, after the desquamation has taken place one time you will often find it taking place again, and this may happen within six or seven weeks afterward. I do not believe the contagion resides in this second desquamation. I do not think we should appoint one man to dictate to the profession in regard to these points.

*Dr. Kime:* As to the duties of the inspector, when there is a case of scarlet fever, for instance, send the inspector to examine the patient and confirm the diagnosis, and if the family or physician need any help, or the physician in charge is not disinfecting the room, and allowing the people to go out, the inspector calls the attention of the Board of Health to this, and has some action taken. Also, when ready to discharge a patient the Board of Health is notified, and they, in order to satisfy themselves, will send the inspector to examine the case.

*Dr. Johnson:* I would like to ask how many physicians, he thinks, will submit to this? We cannot control this until there is some agreement in the minds of the physicians in regard to the spread of these diseases. They go from one sick-room to another wearing the same clothes without any precautions. I would like to know how many physicians change their clothing after visiting scarlet fever cases. Until the doctors make up their minds in regard to such matters we cannot hope to accomplish anything by an inspector.

*Dr. Robinson:* I don't think the duties of the inspector would conflict with the physicians in any way. The physician would notify the Board of Health of cases of diphtheria, scarlet fever, etc., and they would simply send the inspector there, not to interfere with the physician, but simply to see that the patient is properly isolated and the precautions properly carried out, and to see that the family does what the physician has told them to do. The physician has not the authority with a family that a medical inspector would have, as they would do anything the inspector directed, because of the law.

In regard to desquamation, there is no question that there are cases here in Atlanta that have been turned loose while they were still desquamating and while there were questionable bacteria in the throat. We all know that the disease is spread in this way. I remember a case in hospital experience of a child that had diphtheria and the throat had been perfectly clear for a long time, and some two or three weeks afterward it went with other children and two of them were taken with diphtheria. The child went on to New York and came in contact with other children and one or two died from diphtheria. A bacteriological examination was then made of this child's mouth, and it was found loaded with the bacilli. The child had a slight snuffles all the time.

Now, this inspector is also supposed to be sufficient bacteriologist to make these examinations, and it relieves the physician of the responsibility. When the physician is ready to dismiss a case of diphtheria the inspector makes a culture, and if negative he discharges the case, but if not, the physician would certainly agree with him that it should not be discharged.

Cases of scarlet fever are turned loose entirely too soon, and it would be the duty of the inspector to look after these, and I don't think the inspector should antagonize the physician in any way. The Board of Health are practicing physicians and have not time to do this thing, and the city inspectors are not competent for this and are for other purposes. It was my idea that the inspector see that all cases are properly isolated and the instructions carried out. I have seen cases where they were given positive instructions in regard to the isolation of the patient, but they would not carry them out, but if they knew it was the law and that the inspector would see to it, they would conform to the instructions. I cannot see how the inspector would antagonize the physicians or interfere with them in any way.

*Dr. Stirling:* I only want to say a word or two, as I said a good deal two weeks ago on this subject. I feel myself convinced that the Board of Health is insufficient to look after the health of the city, with the inspectors. The inspectors are not medical men, and the Board of Health sits in the office and cannot go out to look after things. We want a man to go between these two, and he should be a medical man; such a man is urgently wanted here and in every city. Dr. Johnson has spoken as if he would be a menace to the physicians. I think he would welcome the health officer and be pleased with the arrangement after a few months. I know this, for I was in this work for three years in England, and I have never seen any difficulty. The whole matter is under the Local Governing Board, sitting in London, who look after the appointing of these men, and they have the entire health of the country in their hands. We have three medical men on the Board of Health here, but on that board there were none at all, and therefore we are that much better off here. I feel quite certain that no one would find a doctor in that position to be objectionable. Dr. Johnson spoke of the difference of opinion as to the time different diseases are held to be contagious. This has been settled sufficiently as to be governed by laws, and I do not think there would be any trouble with a man of ordinary tact and who is received as he should be. I would like very strongly to urge all of the medical profession to put their shoulder to the wheel and shove this through.

*Dr. Visanska:* I think this subject under discussion is extremely theoretical and not at all practical. The theory is very good, and the man would sit in his office and receive a good salary and wait for the physicians to call for him to diagnose cases. I would consider the position as a good thing. I do not believe this method could be put into practice. It would place the officer over the physicians, and it would hurt the medical profession in a good many ways. We would be compelled to call upon this officer to see all these cases. I think the doctors are able to diagnose their own cases, and if they have any doubt they will call in another practitioner.

*Dr. Hutchins:* In regard to those cases of late desquamation as mentioned by Dr. Johnson and Dr. Robinson, if the result of scarlet fever they ought not to be exposed if there is any doubt of their infectiousness. However, I think it is very doubtful whether some of these cases are the result of scarlet fever, but are often probably due to toxic erythema. I know a little girl whose case was diagnosed as scarlet fever and was so considered for a long time, but who has a toxic erythema of the skin. Wherever you have a continued excessive hyperæmia of the skin you will have desquamation following.

As regards the health officer, this method has been used in the large cities, and I think we will have to come to it sooner or later, and I cannot see how it would interfere with the physicians. I think it would be a good help to those men who go around without changing their clothing.

*Dr. Crawford:* If I should be on the Board of Health and this method were adopted I should feel pretty bad. It seems to me that this all comes under the jurisdiction of the board, and there is no need of having the two offices.

*Dr. Kime:* It is the sentiment of the Board of Health that this would interfere not at all with them, but does a work that they cannot do.

*Dr. Crawford:* I would say differently, and abolish the Board of Health and put this man in its stead.

*Dr. Duncan:* I live in the neighborhood of the Boulevard School, and a great deal has been said about the fever there. I have not seen a case that developed in that school. I had a case of scarlet fever on Jackson street, in the neighborhood of the school, but had not been to school but had one or two sisters going to school. The disease came from a broken sewer which was between two houses, and scarlet fever developed in both houses as the result. I have seen it in other families where six or eight would have it, not from contagion but by drinking well water that was contaminated with animal matter. When we eliminate all other sources of contagion we will not have trouble in the schools.

*Dr. Hubbard:* I think that if we got a man to make the bacteriological examinations it would be a good thing for all of us. Also, it would be a good plan to have him superintend the disinfection of the houses. But to get a man to go around and superintend the isolation of every case of contagious disease would be impossible. I think a medical man should be employed for this work.

*Dr. Collins:* In regard to the medical inspector interfering with the physicians, he, to my mind, simply represents the legal side, and he can compel the people to do what the physician cannot get them to do. They will not carry out instructions given by the physician, but if they understand that they are under the law, the instructions will be carried out nine times out of ten where they would not. He is simply backing up the physician with the legal authority.

*Dr. Kime:* What the committee has done and said in this matter has been simply as a "feeler," and if the Society is not going to back us up there is no use for us to fool away our time. Under the best circumstances it will be a hard fight to get the Council to carry this out, as they are men not in sympathy with such work. We want the backing of the Society, and we want you to discharge the committee unless we get this.

*Dr. Stirling:* I agree with Dr. Kime that if we do not have the backing of the Society you should dismiss us. This work is being carried on in cities that are advanced, and Atlanta is supposed to be advancing and it has to come some time, and why not have it now. The expense is really a saving to the city and to the State.

*Dr. Johnson:* I think the committee are retreating too soon. There has been no opposition to the movement. The committee reported for discussion, and we have very religiously discussed it, and I believe, for the good. Now, some have differed, but I have not heard one say but what it was a good movement—that is, the control of the spread of contagious fevers. I cannot see why the committee wants to be dismissed, because there has been no opposition—has been simply a discussion. We have discussed it with the end of finding the best plan of procedure to be adopted. Also, you could hardly find a body of men such as this who would favor the appointment of such an officer unless they knew something of the functions of this officer. We wanted to know this. We wanted to see which was the best way to proceed, and I think this is the sentiment of the Society.

*Dr. Kime:* We have not had a meeting of the committee with the Board of Health, but have only had an informal discussion with the different members of the Board. Of course, my idea before final action, was to meet with the Board of Health and decide upon the duties of the medical inspector, and then have the endorsement of the entire membership of the Society. It would do no good to go before the Council without this.

*Dr. Hutchins:* I move that the committee be continued for further conference with the Board of Health.

Dr. J. M. Crawford was on the program for a paper, but spoke as follows:

I have no paper for the evening, and on account of the hot weather thought it would probably be of interest to some to simply report a few cases of operation for mastoiditis in the last three years. I have in that time operated upon seven cases, with a mortality of one. We all know that inflammation of the mastoid cells is brought on through inflammation beginning with the nose and throat back into the eustachian tube and into the middle ear, and thence into the mastoid cells. It is indicated by pain, swelling, etc., but the swelling is peculiar, in that it throws the ear or pinna downward. However, such a condition can be produced by other than mastoiditis. For instance, a furnacle will cause the same drooping in the auditory canal. You have to be guided by the symptoms of pain, etc., as to whether it is one or the other.

When we have a case of mastoiditis the first thing is to see if we cannot abort it. This has been done on several occasions by puncturing the drum membrane and letting the pus out there.

On November 6, 1896, I had a patient five years of age, and she came to my office with a history that the ear had been suppurating for about three months. I will say just here that most cases come from acute suppurating inflammation of the middle ear; not from chronic but from acute, but this came on about three months after the first suppuration. She presented herself late in the afternoon, and I gave her chloroform and operated. Nothing was needed more than Wilde's incision with a bistoury, and I cut down through the periosteum and the cortex and let the pus out. In many cases nothing more is necessary than the letting out of the pus.

November 26, 1896, I had a patient six years of age who had had scarlet fever, and about one month after this, while in bed, had a suppurating inflammation of the middle ear, and in a few days mastoiditis came on, and I operated on her without an anesthetic, on account of her condition from the fever. With a strong bistoury I cut down through the periosteum and the pus was evacuated.

April, 1897, had another case, aged five years. She had inflammation of the middle ear about one month, and Wilde's incision was made and the pus was evacuated.

These cases were in children from 3 to 5 years of age, and needed nothing more than cutting through the periosteum, as the cortex was necrosed.

In 1897, a man aged 50 had suppurating inflammation of the middle ear, and for some three weeks was treated by some one, and two days before presenting himself to me complained of a pain behind the ear, and there was drooping of the pinna, etc., and I diagnosed it was mastoiditis, and did not think it well to wait longer, but operated upon him, giving ether. The outer cortex was so hard that the bistoury would not cut through, and I broke through with chisel. I kept the patient in bed and dressed twice a day for one month, and he recovered.

The next was in 1898; a man aged about fifty years, had about the same appearance and history, and after inflammation of about one month mastoiditis developed, and under ether made the same operation, cutting through the periosteum and using chisel, and treating for about eight weeks.

Of the other two cases, one was a woman about thirty years of age, and presented herself before there was any suppuration of the middle ear. She complained of ear-ache, and had suffered for several days. I gave laxative and spray to get rid of inflammation of the nose and throat, and told her to return to her home in Fayetteville. She came back in a week, but there was still no suppuration, but there was bulging of the drum membrane and distinct symptoms of mastoiditis. I told her it would be necessary to give chloroform or ether, and operate. However, I found she had heart trouble, and so did not give her anything. I made superficial incision, and then used a small probe and found a small point of necrosis, and then took a curette and made a large opening into the mastoid cells, instead of using the mallet and chisel.

The most interesting case was the last one. Some two months ago Drs. Benson and Odell sent for me to make operation for mastoiditis. I found the patient had been unconscious for two days and a half, and had had suppuration of the middle ear for three or four weeks; had been under the care of a doctor for this trouble, but after getting in bed had not been out to see him. Had been unconscious for two and a half days, and had rigors. I gave ether, and made an opening into the mastoid cells. The patient lived two weeks and one day, and then died. Held post-mortem, and found had general meningitis, and also found had abscess just inside the skull, near the situation of this mastoiditis. This last case, as I said, was the most interesting one. It is quite unusual that the patient dies after a mastoid operation, but I was satisfied that the cause of death was the amount of inflammation and the pus getting into the brain before the operation was made. It was sufficient to set up meningitis. The patient never fully recovered consciousness after the operation. I made no examination with the ophthalmoscope, as patient complained a good deal.

*Dr. W. E. Campbell:* It was not my intention to report any case, but to-day at the College a patient who had had some mastoid trouble expressed a desire to hear this paper, and I asked him to come up. His case is somewhat interesting. About the last of September he was attacked by a very severe pain in the right ear. He was traveling at the time, and going to Macon, he consulted an aurist, who pronounced it an acute inflammation of the middle ear, and made an incision and removed the discharge. He went to Savannah and there had treatment, and the discharge ceased there entirely. Traveling into Alabama in the cold weather, he was seized with severe pain in the ear, and swelling and indications of mastoiditis. The parts pointed at two places in the floor of the external auditory canal. These places bursted and he got some relief. He went to Nashville, and all were in doubt as to diagnosis, and none decided upon mastoiditis. After this he came to Dr. Calhoun with a discharge from these places, and we treated for a week or two with various washes, and with probes we found that the two openings connected. We gave treatment, and under this he got along very well. Finally he developed some acute swelling or inflammation, which I think was due to pressure from the syringe. The Doctor advised operation, which was done the latter part of March. There was nothing particularly of interest in the operation, but upon cutting down he found leading from these openings a small necrosis of bone, and chiseled out the mastoid cells. The parts healed up beautifully. There was no pus except at the fistulous opening, and there seemed to be no connection between the middle ear and the mastoid cells. Since then he has had only the irrigation of the canal, and has gone on to recovery. I don't know that this case is of any particular interest, but during the time that he had consulted the best specialists in these different places none had agreed that it was mastoiditis. Some thought it was glands, but I did not think so, as there were none at that situation.

In regard to Dr. Crawford's paper, I do not think he goes far enough in simply cutting through and breaking down these parts. I think he should go further and curette and remove the contents.

*Dr. Crawford:* I might have gone into the cases more minutely, but on account of time I went over them hurriedly. Speaking of simply opening the parts, I always get out the necrosed tissue which is in the mastoid cells. Nothing is better for opening the outer cortex than the mallet and chisel. Another case which I might have mentioned more minutely was that one patient had mastoiditis on both sides. Another doctor made operation on one side and afterward I made operation on the other. All of them get well where they are made early enough, and made in proper position. There is not any danger when the operation is made properly. It is not good surgery to make Wilde's incision and then leave it, as I have seen it done, and by poulticing, wait for nature to break through the outer cortex. The mallet and chisel should be used and the mastoid cells opened.

In the doctor's case I would have made an opening into the drum membrane and kept it open by Valsalva's method of inflation of the middle ear. He would probably not have had the mastoiditis had this been done. The inflation blows the pus out through the opening in the drum, and this should be kept up until the inflammation passes off.

